# Follow-up of suspected child maltreatment cases treated at a tertiary child protection service facility

**DOI:** 10.1007/s00431-026-06803-y

**Published:** 2026-02-23

**Authors:** Eva Anna Mora-Theuer, Sophie Klomfar, Christoph Krall, Chryssa Grylli, Klara Doppler, Maria Kletecka-Pulker, Sabine Völkl-Kernstock, Gabriel Otterman, Paul Plener, Judit Simon, Susanne Greber-Platzer

**Affiliations:** 1 Forensic Examination Centre for Children and Adolescents, Division of Pediatric Pulmonology, Allergology and Endocrinology, Department of Pediatrics and Adolescent Medicine, Medical University of Vienna, Waehringer Guertel 18-20, Vienna, 1090 Austria; 2 Center for Medical Data Science, Medical University of Vienna, Vienna, Austria; 3 Institute for Ethics and Law in Medicine, Medical University of Vienna, Vienna, Austria; 4 Department of Child and Adolescent Psychiatry, Medical University of Vienna, Vienna, Austria; 5 Barnafrid National Centre On Violence Against Children, Linköping University, Linköping, Sweden; 6 Department of Health Economics, Center for Public Health, Medical University of Vienna, Vienna, Austria; 7 Department of Psychiatry, University of Oxford, Oxford, UK

**Keywords:** Child protection, Non-accidental injury, Community child health, Pediatric A&E and ambulatory care

## Abstract

The victim’s physical and emotional safety are foremost priorities in the clinical care of suspected child maltreatment (CM). The WHO recommends follow-ups, but we lack standardized procedures. This study examined case characteristics in relation to outcomes and follow-up (FU) feasibility at the Forensic Examination Center for Children and Adolescents (FOrensische Kinder- und JugendUntersuchungsStelle, FOKUS), Austria’s first tertiary healthcare service offering regular FUs of CM cases. Suspected CM cases presented to FOKUS between July 2015 and June 2017 were retrospectively analyzed regarding one-year FU outcomes. FU feasibility, timing of FU, and reasons for delay or no FU were investigated. Two groups (FU vs. no FU) were compared. In the FU group, cases discharged from FOKUS services after the initial FU visit were compared with those remaining under FOKUS care regarding child protection (federal child protection service involvement, reports to law enforcement), safety procedures (new CM incidents, new injuries/findings/observations, contact with suspected perpetrator), and therapeutic interventions. Associations between case characteristics (age, gender, FU length, suspected CM type, perpetrator) and the groups were analyzed using logistic regression models. Of 219 cases, FU was feasible in 64%. Inaccessibility of families was the main reason for no FU. Followed patients were younger and perpetrators were more often parents (OR = 2.08, 95% CI 1.10–3.99). Suspected sexual abuse cases and those with new CM incidents were less frequently discharged (OR = 0.31, 95% CI 0.10–0.85 and OR = 0.18, 95% CI 0.05–0.66).

*Conclusion*: CM case characteristics permit personalized, structured FU planning and scheduling to improve safety and continuity of care.

**Table:** 

**What is Known:** • *The WHO recommends regular follow-up (FU) of suspected child maltreatment cases to ensure patient safety and prevent further harm, but standardized procedures are lacking.* • *In Austria, hospital-based child protection teams are legally required, yet systematic FUs and structured safety monitoring are rarely implemented nor legally required.* **What is New:** • *This first CM follow-up study from Austria’s tertiary child protection service (FOKUS) shows that FU was feasible in 64% of cases.* • *The results suggest that case characteristics influence the success of FU. Structured, individualized FU and sufficient resources are essential to enhance patient safety and long-term protection.*

**Supplementary information:**

The online version contains supplementary material available at 10.1007/s00431-026-06803-y.

## Introduction

According to the World Health Organization (WHO), child maltreatment (CM) includes all types of abuse and neglect (physical and/or mental abuse, sexual abuse, physical and/or mental neglect, abandonment and commercial or other exploitation) in relationships of responsibility, trust, or power involving children under 18 years [1]. CM causes actual or potential harm to a child’s health, development, or dignity, and increases lifelong risks of cognitive impairment, mental disorders (e.g., post-traumatic stress disorder, depression), addiction, obesity, cardiovascular disease, cancer, sexually transmitted infections, unwanted pregnancies, and future violent behavior. It also contributes to educational and economic inequalities [1]. The resulting societal burden includes trauma-related costs of healthcare, social and educational services, and productivity losses from poor vocational skills, unemployment, and disability [2, 3].

Ensuring the physical and emotional safety of suspected victims of CM is a top priority in medicine. Health professionals must act to prevent further harm and minimize negative consequences. The WHO recommends careful and regular follow-up (FU) of CM, including monitoring referrals, developing FU plans that specify future contact with the child or caregivers, and steps to be taken if contact is lost. The “WHO Guideline for the Health Sector Response to CM” provides suggestions to identify and prevent recurrent maltreatment, emphasizing safety and risk assessment concerning patients [4]. However, it gives no standardized procedure for conducting FUs or creating individualized FU timelines based on CM type.

International studies recommend frequent FU of CM in healthcare settings [5–10]. FU is more effective when hospital-based child protection teams work through multidisciplinary collaboration [11–13].

### Child protection in Austria

Protecting the best interests of children is a key responsibility of the Austrian legal system. Mandatory reporting requirements exist for suspected CM in healthcare settings. Hospital-based child protection teams, legally implemented since 2004, permit early detection through multidisciplinary expertise [14–20]. Reporting crimes such as death, severe injury, rape, abuse, neglect, torture, or sexual abuse of children, adolescents, or vulnerable adults is standardized for all healthcare professionals [16, 21]; the latter are obliged to promptly report any reasonable suspicion to statutory authorities, including federal child protection services (fCPSs) and law enforcement (LE). Reporting to LE can only be waived if all of the following conditions are met: (1) doing so is in the child’s best interest, (2) the suspect is a close relative, (3) the fCPS has been informed, and (4) a hospital-based child protection team is involved if possible [14, 21–23].

Although Austrian law mandates reporting of suspected CM and requires an initial risk assessment by child protection services, it does not explicitly prescribe WHO-recommended FU procedures such as medical and psychosocial monitoring or long-term care planning [4, 14–16, 22, 23]. In addition, feedback from fCPSs or LE to healthcare facilities on reported cases is uncommon, as these institutions are not legally required to provide updates. Existing publications on hospital-based child protection in Austria have noted that routine FUs of CM cases are rarely performed. While such teams are considered valuable, the absence of long-term FU remains a major structural and procedural shortcoming [17, 20, 24–28].

The Forensic Examination Center for Children and Adolescents (FOrensische Kinder- und JugendUntersuchungsStelle, FOKUS) was established in 2015 to address these shortcomings. FOKUS is Austria’s first tertiary center supporting regional hospital-based child protection in Vienna. It is funded by the Federal Ministry for Social Affairs, Health, Care and Consumer Protection, the Vienna Health Council, and the Medical University of Vienna, and is based at the Department of Pediatrics and Adolescent Medicine [24, 29].

All regional hospital-based child protection teams may report suspected CM to FOKUS, which provides support according to the respective needs:

- Medical and psychological evaluation and the management of suspected CM in the healthcare sector (e.g., physical and forensic examinations, psychological examinations and support, telephone consultations to give guidance about investigations, documentation and reporting)
- Checklist templates for forensic documentation
- Evidence-based guideline information
- Assistance in contacting and collaborating with fCPSs, other social services, LE and public prosecutors
- Lectures and training courses on child protection procedures for all health care professions
- Systematic data collection of CM cases
- FU of suspected CM cases one year after initial reporting to FOKUS

As a tertiary umbrella service, FOKUS complements and supports but does not replace the legally mandated responsibilities of hospital-based child protection teams, fCPSs and LE, which remain primarily responsible for implementing protection measures and ensuring the child’s safety.

As a coordinating service, FOKUS stores non-anonymized patient data in the official medical records. During its first year, FOKUS introduced supplementary structured FU visits aimed to improve diagnostic accuracy, patient safety, and care. These FU visits included evaluation of WHO-recommended components, such as safety and risk assessments, identification of potential recurrent maltreatment, monitoring of implemented protection measures, and assessment of the need for further medical and psychosocial interventions beyond the initial CM episode. Although hospital-based child protection teams are a legal requirement since 2004, FOKUS remains the only healthcare service, in its role as an umbrella organization, to routinely perform FU for CM patients [15, 24].

The WHO and the published literature clearly recommend FU of CM in the healthcare sector. Routine FUs should serve the best interests and safety of victims while remaining feasible for child protection services providing care [4–10]. This study aimed to examine characteristics of suspected CM cases at FU visits, including child protection and safety measures, interventions during the FU period, and the need for further interventions within the tertiary child protection service. Additionally, we assessed the feasibility and procedural challenges of conducting FU visits.

## Methods

### Study design and setting

A retrospective cohort study of suspected CM cases reported to FOKUS during its first two years (1 July 2015–30 June 2017) was performed. Data were collected from the time of the initial case report to FOKUS services through the scheduled FU visit, which was planned one year later.

Suspected CM cases are reported to FOKUS via hospital-based child protection teams, which act independently and initiate FOKUS involvement on a case-by-case basis following consultation and a request for FOKUS support. Depending on case-specific needs, FOKUS involvement can occur at an early stage to support forensic diagnostic examinations or during the clinical course when unanswered questions remain or when most additional investigations have already been completed. Accordingly, the initial abusive events frequently occurred before the first suspicion of CM and prior to the child’s presentation to a hospital. Moreover, the time between the abusive event, emerging suspicion, hospital presentation, and eventual FOKUS involvement varied from immediate to timely or delayed, and in some cases, the timing was unspecific. For this study, the date of the initial report to FOKUS was used as the primary temporal reference point.

FU visits were conducted between 18 June 2016 and 19 February 2020.

### Human ethics and consent to participate declarations

The study received ethics approval (1253/2016) and data protection approvals from the Ethics Committee of the Medical University of Vienna in accordance with the Declaration of Helsinki. This retrospective cohort data analysis lacked any specific patient or public involvement.

### Follow-up visits

FOKUS introduced one-year FU visits as a standardized procedure to monitor the implementation and long-term effectiveness of child protection measures and therapeutic interventions. A one-year interval was chosen pragmatically, allowing sufficient time for initial case management activities coordinated by FOKUS, completion of mandatory reporting, and for prescribed interventions to take effect, while also providing the opportunity to evaluate their effectiveness. For each case, the “FU period” was defined as the time (in days) from the initial report to FOKUS to the completion of the FU visit, reflecting the timing of the FU assessment rather than the duration of ongoing care.

During FU visits, issues occurring after the initial report to FOKUS and up to the FU visit were assessed and documented, including compliance with prescribed child protection measures, therapeutic interventions, and decisions regarding discharge from FOKUS services after FU. Child protection procedures (fCPS involvement and reporting to LE) and therapeutic interventions were assessed longitudinally only in patients who attended a FU visit. Although some information on these aspects may have been available at the time of initial case presentation to FOKUS, systematic and comparable assessment over time was not feasible in patients without a FU visit.

For this purpose, CM patients and their parents or legal guardians were invited to attend the FU visit. As direct access to fCPS or LE records was not available, information on interval events was primarily obtained through structured history-taking with patients or guardians. A standardized FOKUS-specific FU checklist supported and guided all FU assessments (Supplement 1), recording patients’ well-being, child protection procedures (fCPS and LE involvement), safety measures (new CM incidents, suspicious findings or injuries, contact with suspected perpetrators), and therapeutic interventions. Physical and/or psychological examinations were conducted when clinically indicated in the patient’s best interest.

**Fig. 1:**
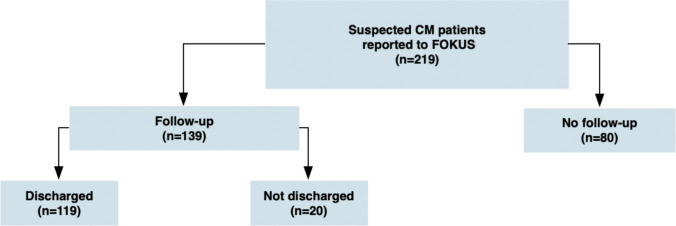
Patient groups

Cases were divided into those for whom FU was feasible (FU group) and those for whom it was not (no-FU group) (Fig. 1). FOKUS patients or their parents or legal guardians, including foster parents or representatives from care institutions or fCPSs, were contacted by phone to schedule a FU appointment one year after the initial report to FOKUS. If inaccessible by phone, a letter with a pre-scheduled visit date was sent. If still unreachable, FOKUS contacted the responsible fCPS for information or filed an official report if the child’s welfare was at risk. FU visits were classified as “briefly delayed” (458–486 days) or “extensively delayed” (> 486 days). Cases with without a FU visit within a maximum FU period of 850 days after the initial report to FOKUS were categorized as no-FU.

All patients and their legal guardians were invited to a personal FU visit. Phone-based FU was only considered when a personal visit was impossible. FU visits were conducted by a pediatrician and/or clinical psychologist, depending on the type of suspected CM and the patient’s initial care. Cases of suspected physical abuse or neglect were followed up by a pediatrician, with a psychologist joining as needed, based on age, stress, prior psychological evaluation, or to ensure dual control. For other CM types without expected physical injury or neglect, a psychologist could conduct the FU alone with physician support if necessary.

### Discharge from FOKUS services

If no further evidence of suspected CM was found during the FU visit, based on structured clinical and psychosocial assessments, the patient was discharged from FOKUS services (“discharged”; Fig. 1). Discharge could indicate that prior interventions and external measures were effective, and ongoing monitoring or interventions by fCPSs or other involved external services could continue as indicated. If the team determined that ongoing care was necessary to ensure the patient’s well-being and safety, further measures were planned, and the patients remained under FOKUS care (“not discharged”; Fig. 1). For these patients, additional medical, psychological, or interagency measures were initiated or coordinated.

### Data management

FU checklist data were merged with case information from the initial FOKUS involvement (such as the date of first report to FOKUS) in a harmonized Microsoft Excel (v16.0) database. We included all suspected CM patients under 18 years reported to FOKUS between 1 July 2015 and 30 June 2017. The actual FU period for each case was derived from these records.

Case characteristics (age, gender, FU period, type of FU visit, suspected CM type, suspected perpetrator), child protection procedures (fCPS involvement, LE reporting), and therapeutic interventions performed during the FU period were analyzed in relation to FU outcomes and the need for care beyond the FU period (“not discharged” vs. “discharged” cases). All therapeutic interventions were recorded, regardless of whether they were conducted within healthcare or other institutions.

Patient safety measures (new CM incidents, suspicious findings or injuries, and contact with suspected perpetrators) arranged during the FU period were analyzed in relation to FU outcomes (“discharged” vs. “not discharged”). The measure “contact with suspected perpetrator” was prioritized over out-of-home placements, as placements did not always ensure safety: patients could maintain contact with the perpetrator, return to unsafe environments during temporary placements, or have unsupervised family visits even in permanent placements.

All data were cleaned before analysis.

### Statistical analysis

Descriptive analyses were performed for the entire cohort and separately for FU and no-FU groups. Categorical variables were compared using Chi-squared or Fisher’s exact test. Quantitative variables were reported as median and interquartile range (IQR) and compared using Mann–Whitney *U*-tests.

For data validation, the association between FU attendance and patient type (index patient vs. sibling/other household children) was analyzed in families with multiple children using a logistic regression mixed model with family as a random intercept.

FU attendance was analyzed in relation to suspected CM type and suspected perpetrator type using logistic regression models. In families with multiple children, one child was randomly selected to avoid dependent observations. Sensitivity analysis was performed using a generalized estimating equation.

In the FU group, associations between discharged cases and case characteristics (age, gender, FU period length, suspected CM and perpetrator type, and interventions during FU) and safety measures (new CM incidents, suspicious findings/injuries, new observations, and contact with suspected perpetrators) were analyzed using logistic regression, again sampling only one sibling per family. Cases labeled “unclear” were treated as unassigned.

Correlations among the most common abuse types (physical abuse, sexual abuse, and neglect) were assessed using Cramer’s phi and Fisher’s exact test.

Survival curves for FU attendance over time since first reporting to FOKUS were plotted overall, by sex, and by CM type. Median times with IQR and log-rank test *p*-values were reported. IBM SPSS, version 29 for MAC, and R, version 4.3.0, were used for analysis.

## Results

### Study population

A total of 219 patients with suspected CM who met the inclusion criteria were reported to FOKUS between 1 July 2015 and 30 June 2017. One patient was reported twice; the second report was excluded from the analysis (Fig. 1).

Of these, 139 patients (63.5%) received a FU visit from FOKUS (FU group). Ninety-nine (71.2%) visits were conducted in person and 40 (28.8%) by phone. Among patients from households with more than one reported child**,** each case was analyzed individually. No difference was observed in FU attendance between index patients (total *n* = 18, FU group *n* = 14 (77.8%)) and their siblings or other children in the same household (total *n* = 29, FU group *n* = 23 (79.3%)) (OR = 8.75, 95% CI 0.024–3201, *p* = 0.472), with consistent results in sensitivity analysis (OR = 1.21, 95% CI 0.87–1.67, *p* = 0.267). Descriptive results for the total cohort and subgroups are shown in Table 1.

**Table 1:** Study cohort characteristics

	Total (***n*** = 219)		FU group (***n*** = 139)		No-FU group (***n*** = 80)			
Gender	*n*	%	*n*	%	*n*	%	OR (95% CI)	*p*
Male	91	42	59	42	32	40	-	0.833
Female	128	58	80	58	48	60	-	-
Suspected type of CM*	*n*	%	*n*	%	*n*	%	OR (95% CI)	*p*
Physical abuse	89	41	62	45	27	34	1.72 (0.93–3.24)	0.087
Sexual abuse	101	46	57	41	44	55	0.64 (0.36–1.16)	0.144
Psychological abuse	9	4	3	2	6	8	0.36 (0.07–1.53)	0.176
Neglect	47	21	34	24	13	16	1.28 (0.62–2.77)	0.514
Fabricated or induced illness	6	3	3	2	3	4	0.63 (0.11–3.46)	0.571
Unknown course of injury	26	12	14	10	12	15	0.66 (0.27–1.60)	0.347
Suspected type of perpetrator	*n*	%	*n*	%	*n*	%	OR (95% CI)	*p*
Parent yes	110	50	78	56	32	40	2.08 (1.10–3.99)	**0.025**
Parent no	77	35	37	27	40	50	-	-
Parent unknown	32	15	24	17	8	10	3.33 (1.31–9.34)	**0.015**

^*^There were patients with more than one type of suspected CM

*n.a.* not applicable

The mean age of the cohort was 9 years (SD = 5.4). Age differed significantly between FU (mean 8 years; SD = 5.0) and no-FU groups (mean 11 years; SD = 5.7) (*p* < 0.001), whereas gender distribution did not differ (males 42%).

The most common types of suspected CM were sexual abuse (*n* = 101, 46%; FU group: *n* = 57, 41%), physical abuse (*n* = 89, 41%; FU group: *n* = 62, 45%), and neglect (*n* = 47, 22%; FU group: *n* = 34, 24%). Some patients were suspected of experiencing multiple types of CM.

FU attendance was not associated with CM type. However, patients with a suspected parent perpetrator had higher odds of attending FU (OR = 2.08 (95% CI 1.10–3.99) *p* = 0.025), as did cases for whom the presence of a parent perpetrator was unknown (OR = 3.33 (95% CI 1.31–9.34) *p* = 0.015). Frequencies and logistic regression results are shown in Table 1.

In the total cohort but especially in the FU group, CM types showed no correlation between physical abuse and neglect (phi = 0, *p* = 1; phi =  − 0.11, *p* = 0.238, respectively), but strong negative correlations between sexual abuse and physical abuse (phi =  − 0.41, *p* < 0.001; phi =  − 0.4, *p* < 0.001) and between sexual abuse and neglect (phi =  − 0.26, *p* < 0.001; phi =  − 0.24, *p* = 0.005). Bar charts for each combination are provided in Supplement 2.

### Follow-up results

Analyses of child protection procedures (fCPS involvement and reports to LE) and therapeutic interventions during the FU period were performed only in the FU group. Of the 139 patients who attended a FU visit, 116 (84%) had documented fCPS involvement, and 66 (48%) were reported to LE. Therapeutic interventions were documented for 95 patients (69%).

Initial suspected physical abuse was associated with higher odds of fCPS involvement (OR = 7.34, 95% CI 1.92–48.26, *p* = 0.011) and lower odds of receiving therapeutic intervention prior to the initial FU visit (OR = 0.39, 95% CI 0.15–0.97, *p* = 0.045). Suspected sexual abuse increased the odds of receiving therapeutic intervention prior to the initial FU visit more than threefold (OR = 3.49, 95% CI 1.31 to 10.46, *p* = 0.017). Effects for each abuse type on interventions are detailed in Supplement 3.

### Parent(s) as suspected perpetrator(s)

Patients with a suspected parent perpetrator were significantly younger (mean 7 years, SD = 4.4) than those with non-parent perpetrators (mean 12 years, SD = 5.4, *p* = 0.001). Patients with unknown perpetrators were aged on average 7 years (SD = 5.5). Those with suspected parent perpetrators were more often subjected to physical abuse and neglect (*p* = 0.004 and *p* < 0.001; FU group *p* = 0.02 and 0.005, respectively). Details are shown in Table 2.

**Table 2:** Results: “parent as suspected perpetrator” analysis

	Suspected parent perpetrator in entire cohort						
	Yes (***n*** = 110)		No (***n*** = 77)		Unknown (***n*** = 32)		
Type of CM*	*n*	%	*n*	%	*n*	%	*p*
Physical abuse	55	50	28	36	6	19	**0.04**
Sexual abuse	44	40	44	57	13	41	0.07
Psychological abuse	4	4	4	5	1	3	n.a
Neglect	35	32	7	9	5	16	** < 0.001**
Fabricated or induced illness	6	5	0	0	0	0	n.a
Unknown course of injury	6	5	7	9	13	41	n.a
	**Suspected parent perpetrator in the FU group**						
	**Yes** **(*n* = 78)**		**No** **(*n* = 37)**		**Unknown** **(*n* = 24)**		
Type of CM*	*n*	%	*n*	%	*n*	%	*p*
Physical abuse	41	53	16	43	5	21	**0.02**
Sexual abuse	28	36	19	51	10	42	0.3
Psychological abuse	2	3	1	3	0	0	n.a
Neglect	27	35	3	8	4	17	**0.005**
Fabricated or induced illness	3	4	0	0	0	0	n.a
Unknown course of injury	2	3	3	8	9	8	n.a
Interventions	*n*	%	*n*	%	*n*	%	*p*
fCPS involved	72	94	30	83	14	70	0.01
Reported to LE	36	48	25	69	5	25	0.005
Therapeutic intervention during the FU period	48	65	30	88	17	81	0.03

^*^There were patients with more than one type of suspected CM

*n.a.* not applicable

Cases with unknown perpetrators were significantly less likely to be reported to LE (OR = 0.151, 95% CI 0.038–0.518, *p* = 0.004). Frequencies and significance analyses are provided in Supplement 4.

### Discharged cases

The overall discharge rate was significantly lower for cases of suspected sexual abuse (OR = 0.31, 95% CI 0.10–0.85, *p* = 0.028). The reduction in discharge was particularly pronounced when the perpetrator was not a parent or not known (OR = 0.19, 95% CI 0.04–0.73, *p* = 0.023), whereas no reduction in discharge was observed when perpetrators were parents (OR = 0.65, 95% CI 0.17–2.54, *p* = 0.53). No other CM types, gender, or age significantly affected discharge status. Discharged patients were on average 8 years old (SD = 4.9) and non-discharged patients 9 years old (SD = 5.4) (*p* = 0.218). The median time from initial case report to the actual FU visit (FU period) was 577 days (IQR 396–847) for discharged patients and 567 days (IQR 462–840) for non-discharged patients (*p* = 0.591).

New CM incidents, suspicious findings/injuries, and new observations during FU were significantly negatively associated with discharge (OR = 0.18, 95% CI 0.05–0.66, *p* = 0.008; OR = 0.18, 95% CI 0.05–0.58, p = 0.004; OR = 0.194, 95% CI 0.06–0.60, *p* = 0.004, respectively). Frequencies and significant results are listed in Table 3.

**Table 3:** Results: discharge analysis

	Cases after FU visit					
	Discharged (*n* = 119)		Not discharged (*n* = 20)			
Gender	*n*	%	*n*	%	OR (95% CI)	*P*
Male	54	92	5	8	-	-
Female	65	81	15	19	2.13 (0.75–7.01)	0.179
Suspected type of CM – suspected perpetrator no parent/unknown	*n*	%	*n*	%	OR (95% CI)	*p*
Physical abuse	19	90	2	10	2.46 (0.80–24.21	0.135
Sexual abuse	18	62	11	38	0.31 (0.04–0.0.73	**0.023**
Psychological abuse	1	100	0	0	n.a	
Neglect	7	100	0	0	n.a	
Fabricated or induced illness	0	0	0	0	n.a	n.a
Unknown course of injury	11	92	1	8	3.66 (0.60–70.9)	0.240
Suspected type of CM – suspected perpetrator parent	*n*	%	*n*	%	OR (95% CI)	*p*
Physical abuse	37	90	4	10	1.23 (0.23–5.17)	0.764
Sexual abuse	26	93	2	7	0.65 (0.17–2.54)	0.527
Psychological abuse	2	100	0	0	n.a	0.931
Neglect	24	89	3	11	0.912 (0.23–4.57)	0.205
Fabricated or induced illness	3	100	0	0	n.a	n.a
Unknown course of injury	2	100	0	0	n.a	0.905
New incidents of CM	*n*	%	*n*	%	OR (95% CI)	*p*
Yes	11	65	6	35	0.18 (0.05–0.66)	**0.008**
No	88	88	12	12	-	-
Unknown	20	91	2	9	-	-
New suspicious findings/injuries	*n*	%	*n*	%	OR (95% CI)	*p*
Yes	14	64	8	36	0.18 (0.05–0.58)	**0.004**
No	67	91	7	9	-	-
Unknown	38	88	5	12	-	-
New suspicious observations	*n*	%	*n*	%	OR (95% CI)	*p*
Yes	16	64	9	36	0.19 (0.06–0.60)	**0.004**
No	64	90	7	10	-	-
Unknown	39	91	4	9	-	-
Contact to suspected perpetrator	*n*	%	*n*	%	OR (95% CI)	*p*
Yes	59	91	6	9	2.63 (0.7–9.72)	0.143
No	19	79	5	21	-	-
Unknown	41	82	9	18	-	-
Suspected type of perpetrator	*n*	%	*n*	%	OR (95% CI)	*p*
Parent yes	72	92	6	8	4.05 (1.26–14.40)	**0.002**
Parent unknown	19	79	5	21	1.28 (0.37–4.78)	0.705
Parent no	28	76	9	24		
fCPS involved	*n*	%	*n*	%	OR (95% CI)	*p*
Yes	102	88	14	12	1.85 (0.45–6.02)	0.342
No	13	76	4	24	-	-
Reported to LE	*n*	%	*n*	%	OR (95% CI)	*p*
Yes	51	77	15	23	0.2 (0.04–0.67)	**0.017**
No	62	95	3	5	-	-
Unclear	6	75	2	25	-	-
Therapeutic interventions during the FU period	*n*	%	*n*	%	OR (95% CI)	*p*
Yes	77	81	18	19	0 (n.a.)	0.99
No	33	97	1	3	-	-
Unclear	9	90	1	10	-	-

*n.a.* not applicable

### Timing of follow-up visits

**Fig. 2:**
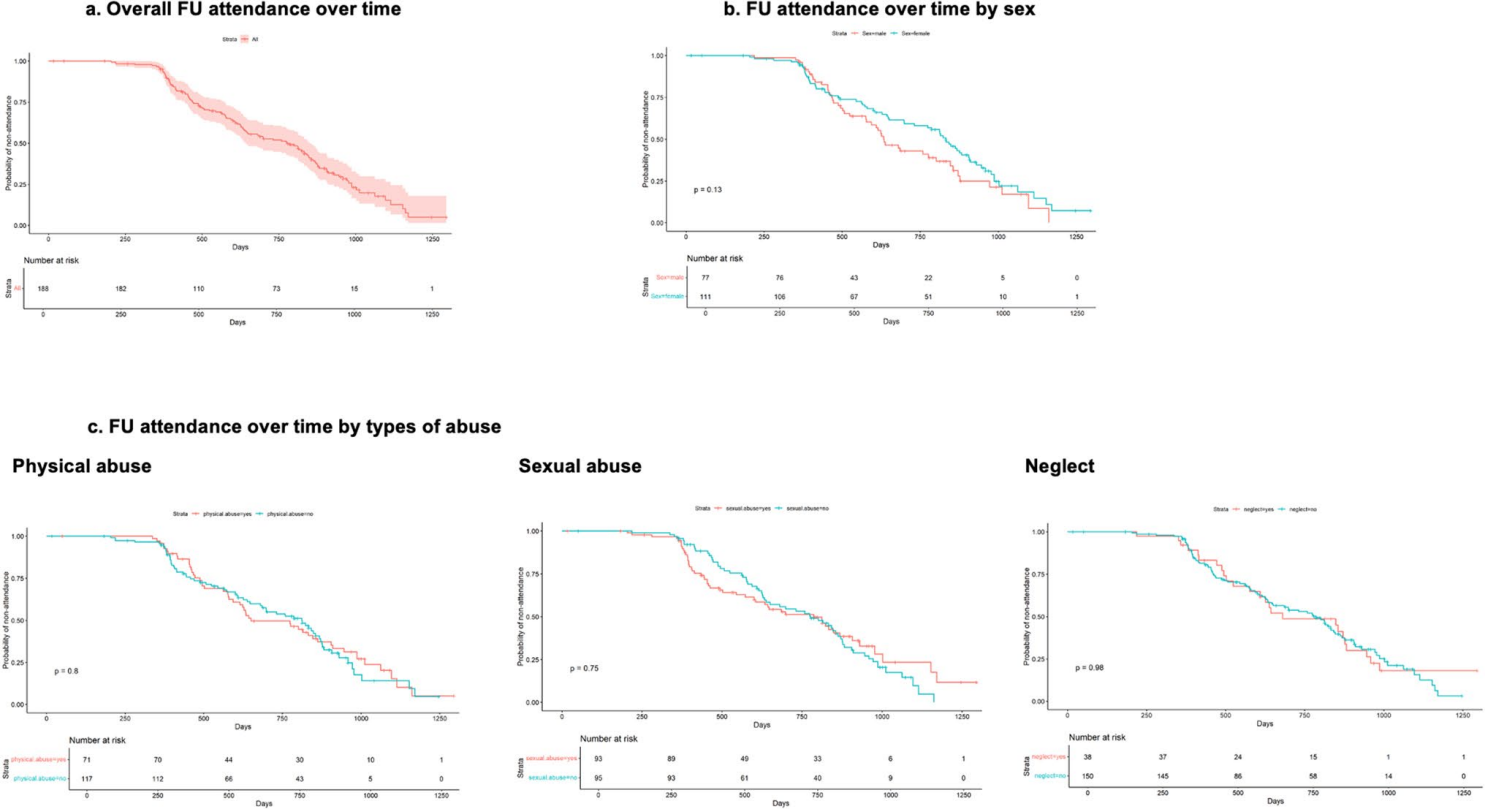
FU times by sex and CM type

Most FU visits (*n* = 91, 65%) were delayed. Twelve visits (9%) occurred early (≤ 356 days after initial FOKUS presentation), 36 (26%) on time (366–457 days), 7 (5%) somewhat delayed (458–486 days), and 84 (60%) extensively delayed (≥ 487 days). Median FU time was 587 days (IQR 502–647). Sex and abuse type did not influence FU timing (Fig. 2). Median FU times by sex and abuse type with log-rank tests are listed in Supplement 5.

The most common reason for long delays was limited professional resources at FOKUS (*n* = 53, 65%), followed by unresponsive legal guardians (*n* = 15, 19%), unknown reasons (*n* = 8, 10%), and other reasons (*n* = 5, 6%).

### Reason for no FU

The main reason for no FU was unreachable patients or no-shows for scheduled appointments (*n* = 47, 60%). Other reasons included refusal by the patient or legal guardian (*n* = 18, 23%), initial suspicion unconfirmed (*n* = 8, 10%), relocation (*n* = 5, 6%), and unknown reasons (*n* = 2, 1%).

## Discussion

FUs of suspected CM patients are infrequent in the healthcare sector. We retrospectively analyzed CM cases managed at Austria’s first tertiary child protection service, FOKUS, and assessed the feasibility and outcomes of FUs, child protection measures, therapeutic interventions during the FU period, and the need for continued intervention beyond FU for patients who could not be discharged.

The type of CM was not associated with the feasibility or timing of the FOKUS FU visits. The most common reason for no-FU was inaccessibility. Given that fCPSs, LE, and other authorities are not obligated to report further case progress to the original healthcare providers, FU of CM cases in the healthcare sector remains suboptimal. Since professional FU is strongly recommended by the WHO, strategies to improve the situation are warranted.

Patients who attended FUs were significantly younger and attendance was higher when the suspected perpetrator was a parent. This raises several questions: could FU rates be improved for patients older than six years or when the suspected perpetrator is not a parent? Could FUs be more effective if scheduled closer to the initial presentation, with prior planning and clear communication to guardians?

Higher FU rates in cases of suspected parent perpetrators may be due to fCPS involvement: 94% of these cases involved fCPS, which may have facilitated attendance although custody was permanently withdrawn only in a few instances. If these factors are relevant, characteristics of CM (type of abuse, suspected perpetrator, patient age) may help in improving FU processes. fCPS involvement appears to positively influence FU attendance, suggesting that structured cross-sector collaboration could enhance FU rates.

Cases of suspected sexual abuse were less likely to be discharged after FU than other types of CM when suspected perpetrators where not parents or unknown, indicating a continued need for care. This suggests that FU procedures might require adaptation for sexual abuse to better address specific needs and improve the quality of child protection.

Involving patients in anticipatory planning of regular and long-term FUs could prevent false expectations regarding the number of visits, length of the FU period, therapeutic interventions, and required child protection measures. Since new incidents of CM, suspicious findings/injuries, and observations during FU were negatively correlated with discharge, scheduling FUs closer to the initial presentation could be beneficial.

The likely benefit of this approach is also emphasized by our data, showing that during the initial FOKUS FU periods, more therapeutic interventions were performed in cases of sexual abuse. The probability of receiving a therapeutic intervention was threefold higher for suspected sexual abuse than for other types of abuse. On the other hand, cases of suspected physical abuse were less likely to have received therapeutic intervention during the FU period. However, suspected physical abuse was significantly associated with a greater likelihood of fCPS involvement, presumably because parents were more frequently suspected perpetrators, necessitating the involvement of fCPS. Suspected physical abuse cases were not less likely to be discharged from FOKUS services after FU had occurred. This may indicate that patients with suspected physical abuse were appropriately referred to and cared for by the fCPSs. Thus, an adjustment of FOKUS services, which had referred those cases to fCPSs, does not seem necessary.

Currently we lack published guidelines on implementing, scheduling, and performing FUs for CM. FUs should prioritize victim safety while remaining feasible for healthcare-based child protection services [4]. FUs required substantial staff effort, as many families had to be contacted multiple times through various channels to determine their whereabouts a year later. During its early years, the FOKUS team was gaining experience in engaging families, which may have contributed to delays. Adequate professional resources and a stable, experienced multidisciplinary team are essential to manage the logistical challenges of FUs effectively. The published literature emphasizes the importance of resources for effective hospital-based child protection teams, while limitations may constrain FU activities, as seen in Austria [8, 25–27, 30, 31]. Despite these efforts, nearly 40% of cases could not be followed up due to inaccessible families, reflecting both practical and structural factors, including Austrian legal regulations.

A specialized institution as FOKUS would benefit from a stable, experienced multidisciplinary team and sufficient professional resources to address logistical challenges, improve scheduling, and enhance FU effectiveness.

The following should be considered when planning FU visits from a specialized unit:

- Focus on cases without fCPSs involvement, the alleged perpetrator is not a parent, and patients are six years or older.
- Schedule FU visits individually in advance.
- Communicate FU plans to patients and guardians in a timely manner and obtain consent as per legal requirements.
- Individual contingency plans for inaccessible patients.
- Consider that patients with suspected sexual abuse may require long-term FUs.

The study presents preliminary FU data from a newly established service facility with no prior experience in implementing, planning, or scheduling FUs for suspected CM in Austria. The ideal FU period for CM cases remains undetermined and warrants further research. Applicability of the results to child protection measures and interventions is limited because we lacked FU information for nearly 40% of the cohort.

## Conclusion

Certain areas should be improved to increase FU rates and the quality of child protection in terms of patient compliance, long-term needs, and safety. These include establishing a stable, experienced multidisciplinary team ensuring sufficient professional resources, focus on cases of a non-parental suspected perpetrator, and patients six years of age or older. FU visits should be arranged individually in advance with the patients and their guardians. Contingency plans for inaccessible patients should be developed in accordance with local regulations. Cases of sexual abuse will need FU for a longer period of time.

## Supplementary information

Below is the link to the electronic supplementary material.

ESM 1 (PDF 549 KB)

ESM 2 (PDF 193 KB)

ESM 3 (PDF 75.6 KB)

ESM 4 (PDF 72.0 KB)

ESM 5 (PDF 67.4 KB)

## Data Availability

The datasets generated and analyzed during the current study are not publicly available due to the sensitive nature of the data and ethical restrictions, but can be provided on reasonable request.

## Abbreviations

CM: Child maltreatment
fCPSs: Federal child protection services
FOKUS: Forensische Kinder- und Jugenduntersuchungsstelle (Forensic Examination Center for Children and Adolescents)
FU: Follow-up
LE: Law enforcement
WHO: World Health Organization
