# Supplementary material for: Follow-up of suspected child maltreatment cases treated at a tertiary child protection service facility
Source: Eur J Pediatr. 2026 Feb 23;185(3):147. doi: 10.1007/s00431-026-06803-y (PMC12929231; doi:10.1007/s00431-026-06803-y)
Supplement: Supplementary file 1 — (PDF 549 KB) [file 431_2026_6803_MOESM1_ESM.pdf]

**European Journal of Pediatrics "*Follow-up of suspected child maltreatment cases treated at a tertiary child protection service facility*"**

*Mora-Theuer E .A., Klomfar S., Krall C., Grylli .C, Doppler K., Kletecka-Pulker M., Völkl-Kernstock S., Otterman G., Plener P., Simon J, Greber-Platzer S.*[*]

## Supplement 1

Follow-up questionnaire - V. 1.1 (status 01/2018)

UNIVERSITÄTSKLINIK FÜR KINDER- UND JUGENDHEILKUNDE MEDIZINISCHE UNIVERSITÄT WIEN

### Follow-up checklist

Name of the child: ______________________

Date of birth: __________ ☐ male ☐ female

Address: ______________________

Patient label with address, if applicable

☐ In person visit ☐ Telephone visit by: ______________________

☐ **One year FU** ☐ **Other FU** **Date of FU visit:** __________

**Reason for delay of the appointment:** ______________________

▸ **Child is brought to the visit by** (Name, Address, Phone, Relationship to the child):

▸ **Current legal guardian** (Name, Address, Phone, Relationship to the child):

▸ **Current residential situation / main place of residence**:

▸ **Maintained contact with suspected perpetrator:**

▸ **Maintained contact with mother / father of the child / person of trust:**

▸ **Current condition** (e.g. (stress-) symptoms and developmental conspicuities) ☐ see additional documentation

Information on the current situation stated by: ______________

▸ **Development in the social environment, changes in kindergarten / school**

▸ **New incidents of maltreatment / abuse / neglect?**

▸ **New suspicious findings/injuries** (→ Body diagram on page 2): F

☐ photo documentation

↳ where archived:

☐ Persistent injuries? Description: ______________________

▸ **New suspicious observations during the FU appointment** ((behavioural problems, contact with the caregiver, signs of neglect):

▸ **Measures implemented to date / others:**

☐ No measures

☐ Child protection services (CPSs) involved - responsible office: ______________ When?

By whom: ______________ Responsible regional office / person: ______________

Involvement of CPSs: ☐ still involved ☐ closed

☐ Report to law enforcement filed when?: __________ by whom: __________ File number: __________

Criminal proceedings:

☐ yes ☐ no if yes: ☐ ongoing ☐ closed; desicion:

Process support provided ☐ yes ☐ no by whom: ______________

▸ **Further measures and resources:**

☐ Therapeutic interventions have taken place? Where? ______________ Since when? ______ Until

Follow-up questionnaire - V. 1.1 (status 01/2018)

UNIVERSITÄTSKLINIK FÜR KINDER- UND JUGENDHEILKUNDE MEDIZINISCHE UNIVERSITÄT WIEN

☐ Other consultations / interventions / measures taken palce (e.g. psychiatry, supportive measures) ______________

▸ **Further planned measures:**

▸ **FOKUS consultation completed?** ☐ **Yes** ☐ **No**

▸ **Miscellaneous:**

▸ **Injury documentation / securing evidence** (Please mark type, description, localisation):

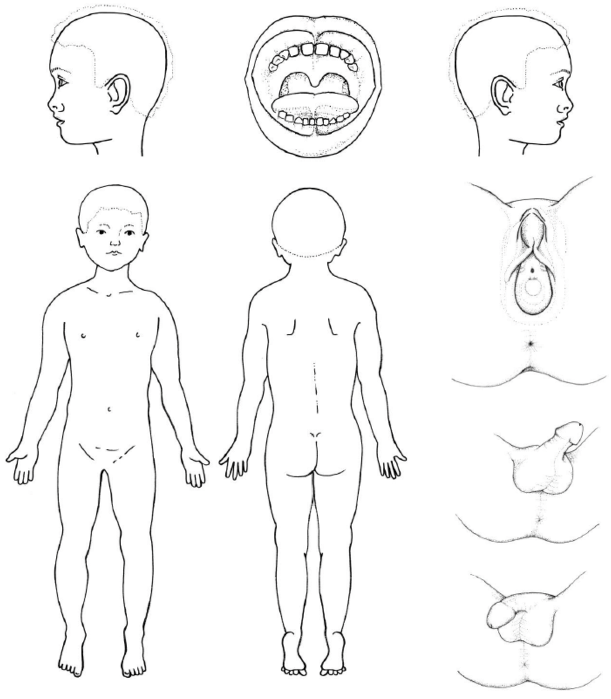

Date: ______________ Name u. signature MD: ______________________

Name in block letters

Contact details (office, phone, email or pager): ______________________

* Corresponding author: susanne.greber-platzer@meduniwien.ac.at, Forensic Examination Centre for Children and Adolescents, Division of Pediatric Pulmonology, Allergology and Endocrinology, Department of Pediatrics and Adolescent Medicine, Comprehensive Center Pediatrics, Medical University of Vienna, Austria
