# Supplementary material for: Follow-up of suspected child maltreatment cases treated at a tertiary child protection service facility
Source: Eur J Pediatr. 2026 Feb 23;185(3):147. doi: 10.1007/s00431-026-06803-y (PMC12929231; doi:10.1007/s00431-026-06803-y)
Supplement: Supplementary file 2 — (PDF 193 KB) [file 431_2026_6803_MOESM2_ESM.pdf]

European Journal of Pediatrics ***"Follow-up of suspected child maltreatment cases treated at a tertiary child protection service facility"***

*Mora-Theuer E .A., Klomfar S., Krall C., Grylli .C, Doppler K., Kletecka-Pulker M., Völkl-Kernstock S., Otterman G., Plener P., Simon J, Greber-Platzer S.**

Supplement 2

Correlations between types of child maltreatment within the entire cohort

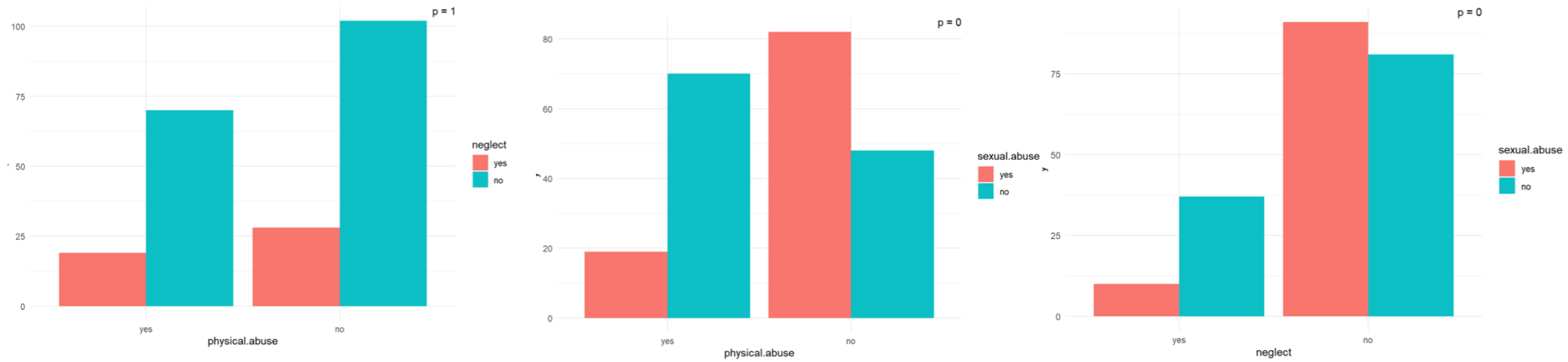

Correlations between types of child maltreatment in the FU-group

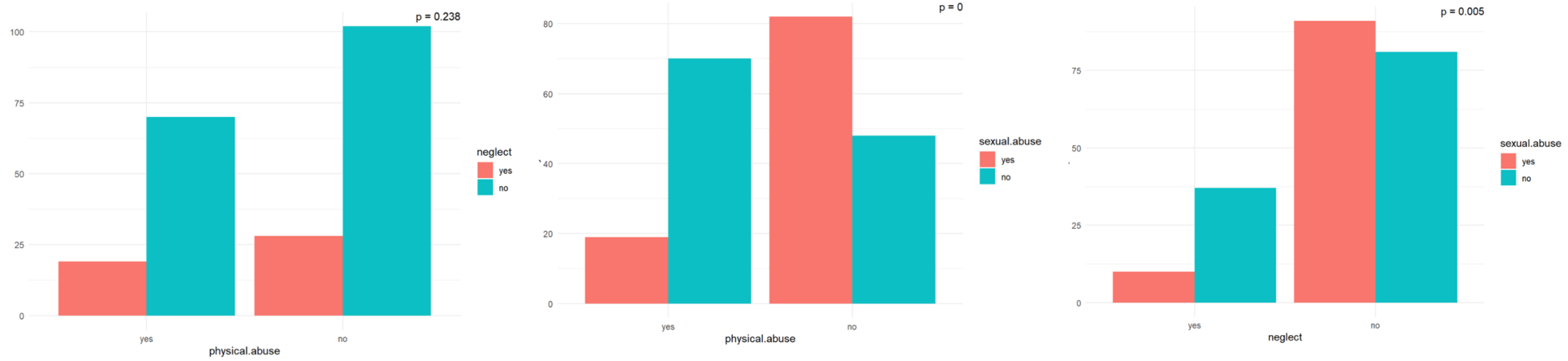

* Corresponding author: susanne.greber-platzer@meduniwien.ac.at, Forensic Examination Centre for Children and Adolescents, Division of Pediatric Pulmonology, Allergology and Endocrinology, Department of Pediatrics and Adolescent Medicine, Comprehensive Center Pediatrics, Medical University of Vienna, Austria
