# Supplementary material for: Follow-up of suspected child maltreatment cases treated at a tertiary child protection service facility
Source: Eur J Pediatr. 2026 Feb 23;185(3):147. doi: 10.1007/s00431-026-06803-y (PMC12929231; doi:10.1007/s00431-026-06803-y)
Supplement: Supplementary file 3 — (PDF 75.6 KB) [file 431_2026_6803_MOESM3_ESM.pdf]

Mora-Theuer E .A., Klomfar S., Krall C., Grylli .C, Doppler K., Kletecka-Pulker M., Völkl-Kernstock S., Otterman G., Plener P., Simon J, Greber-Platzer S.[*]

Supplement 3

Effects of the three most common types of CM on the probability of child protection procedures (fCPS involved, report to LE) and therapeutic interventions during the FU period. Absolute and relative frequencies of patients for each intervention are listed by the presence or absence of each type of CM. ORs are derived from a logistic regression model sampling only one child per family.

	CPS involved			
**Suspected type of CM***	**No**	**Yes**	**OR (95% CI)**	**p**
Physical abuse n(%)	57 (74)	59 (95)	7.337 (1.924 to 48.262)	0.011*
Sexual abuse n(%)	73 (89)	43 (75)	0.374 (0.12 to 1.071)	0.074
Neglect n(%)	85 (81)	31 (91)	1.488 (0.435 to 6.879)	0.56
	**Reported to LE**			
**Suspected type of CM***			**OR (95% CI)**	**p**
Physical abuse	33 (43)	33 (53)	1.663 (0.767 to 3.663)	0.201
Sexual abuse	37 (45)	29 (51)	0.822 (0.328 to 2.058)	0.674
Neglect	48 (46)	18 (53)	1.305(0.605 to 2.842)	0.499
	**Therapeutic intervention during FU period****			
**Suspected type of CM***			**OR (95% CI)**	**p**
Physical abuse	59 (77)	36 (58)	0.385 (0.148 to 0.969)	**0.045**
Sexual abuse	47 (57)	48 (84)	3.486 (1.311 to 2.059)	**0.017**
Neglect	71 (68)	24 (71)	1,418 (0.486 to 3.861)	0.504

* There were patients with more than one type of suspected CM. Therefore, the total number of suspected CM types exceeds the total number of patients.

** For 8 patients (5.8%) it was unclear whether a report to LE was filed. For 10 patients (7.1%) it was unclear whether they had received therapeutic interventions during the FU period.

* Corresponding author: susanne.greber-platzer@meduniwien.ac.at, Forensic Examination Centre for Children and Adolescents, Division of Pediatric Pulmonology, Allergology and Endocrinology, Department of Pediatrics and Adolescent Medicine, Comprehensive Center Pediatrics, Medical University of Vienna, Austria
