# Supplementary material for: Follow-up of suspected child maltreatment cases treated at a tertiary child protection service facility
Source: Eur J Pediatr. 2026 Feb 23;185(3):147. doi: 10.1007/s00431-026-06803-y (PMC12929231; doi:10.1007/s00431-026-06803-y)
Supplement: Supplementary file 4 — (PDF 72.0 KB) [file 431_2026_6803_MOESM4_ESM.pdf]

European Journal of Pediatrics “*Follow-up of suspected child maltreatment cases treated at a tertiary child protection service facility*”

*Mora-Theuer E .A., Klomfar S., Krall C., Grylli .C, Doppler K., Kletecka-Pulker M., Völkl-Kernstock S., Otterman G., Plener P., Simon J, Greber-Platzer S.**

Supplement 4

Effects of suspected perpetrator on the probability of child protection procedures (CPS involved, report to LE) and therapeutic interventions during the FU period.

	CPS involved			
**Suspected type of perpetrator**	**n**	**%**	**OR (95% CI)**	**p**
Parent yes	72	94	2.446 (0.677 to 9.212)	0.17
Parent no	30	83	-	
Parent unknown	14	30	0.462 (0.12 to 1.755)	0.252
	**Reported to LE**			
**Suspected type of perpetrator**	**n**	**%**	**OR (95% CI)**	**p**
Parent yes	36	48	0.420 (0.159 to 1.044)	0.068
Parent no	25	69	-	
Parent unknown	5	25	0.151(0.038 to 0.518)	**0.004**
	**Therapeutic intervention during FU period****			
**Suspected type of perpetrator**	**n**	**%**	**OR (95% CI)**	**p**
Parent yes	48	65	0.344 (0.091 to 1.055)	0.081
Parent no	30	88	-	
Parent unknown	17	81	0.821 (0.16 to 4.615)	0.811

* There were patients with more than one type of suspected CM. Therefore, the total number of suspected CM types exceeds the total number of patients.

** For 8 patients (5.8%) it was unclear whether a report to LE was filed. For 10 patients (7.1%) it was unclear whether they had received therapeutic interventions during the FU period.

* Corresponding author: susanne.greber-platzer@meduniwien.ac.at, Forensic Examination Centre for Children and Adolescents, Division of Pediatric Pulmonology, Allergology and Endocrinology, Department of Pediatrics and Adolescent Medicine, Comprehensive Center Pediatrics, Medical University of Vienna, Austria
