# Supplementary material for: Follow-up of suspected child maltreatment cases treated at a tertiary child protection service facility
Source: Eur J Pediatr. 2026 Feb 23;185(3):147. doi: 10.1007/s00431-026-06803-y (PMC12929231; doi:10.1007/s00431-026-06803-y)
Supplement: Supplementary file 5 — (PDF 67.4 KB) [file 431_2026_6803_MOESM5_ESM.pdf]

European Journal of Pediatrics **“*Follow-up of suspected child maltreatment cases treated at a tertiary child protection service facility*”**

*Mora-Theuer E .A., Klomfar S., Krall C., Grylli .C, Doppler K., Kletecka-Pulker M., Völkl-Kernstock S., Otterman G., Plener P., Simon J, Greber-Platzer S.**

Supplement 5

Median time to FU attendance for sexes and the most common types of CM (physical abuse, sexual abuse and neglect) and log-rank test results.

	Median time in days		
**Sex**	**Median (95% CI)**	**HR (95% CI)**	**p**
Female<br>Male	636 (494 to 633)<br>550 (469 to 33)	0.7713 (0.528 to1.127)	0,134
**Type of CM**	**Median (95% CI)**	**HR (95% CI)**	**p**
Physical abuse<br>Yes<br>No	 604 (502 to 774)<br>567 (458 to 699)	 <br>1.176 (0.807 to 1.713)	 <br>0.803
Sexual abuse<br>Yes<br>No	 458 (414 to634)<br>633 (580 to 801)	 <br>0.7 (0.481 to 1.017)	 <br>0.752
Neglect<br>yes<br>no	 609 (494- to 55)<br>580 (487 to 685)	 <br>0.926 (0.59 to 1.1453)	 <br>0.981

* Corresponding author: susanne.greber-platzer@meduniwien.ac.at, Forensic Examination Centre for Children and Adolescents, Division of Pediatric Pulmonology, Allergology and Endocrinology, Department of Pediatrics and Adolescent Medicine, Comprehensive Center Pediatrics, Medical University of Vienna, Austria
